# An unusual trilogy: a case of comorbid aHUS, Fabry disease, and hypertrophic cardiomyopathy

**DOI:** 10.3389/fmed.2026.1700541

**Published:** 2026-01-30

**Authors:** Qian Li, Lichun Yu, Jing Wang, Zhenle Yang, Yanhua Duan, Junhui Zhen, Shuzhen Sun

**Affiliations:** 1 Department of Pediatric Nephrology and Rheumatism and Immunology, Shandong Provincial Hospital Affiliated to Shandong First Medical University, Jinan, China; 2 Department of Nuclear Medicine, The First Affiliated Hospital of Shandong First Medical University, Jinan, China; 3 Department of Pathology, School of Basic Medical Sciences, Shandong University, Jinan, Shandong, China

**Keywords:** atypical hemolytic uremic syndrome, chronic kidney disease, Fabry disease, hypertension, hypertrophic cardiomyopathy

## Abstract

A 7-year-old boy was admitted to the hospital for abdominal pain, vomiting, and edema. Examinations revealed microvascular hemolytic anemia, thrombocytopenia, acute kidney injury, and hypocomplementemia. He was diagnosed with atypical hemolytic uremic syndrome (aHUS), and treatment was initiated with a methylprednisolone pulse, followed by cyclophosphamide, mycophenolate mofetil, and fresh frozen plasma infusion, leading to remission. At the age of 12, he developed numbness in his fingers and pain in his toes while being febrile. At the age of 17, he presented with aggravated toe pain, renal impairment (creatinine concentration of 156 μmol/L; eGFR of 38.4 mL/min/1.73 m^2^), and remarkable left ventricular hypertrophy accompanied by obstruction of the left ventricular outflow tract. Screening for Fabry disease (FD) revealed a decrease in alpha-galactosidase A (*α*-GalA) activity <1.00 μmol/L/h, along with the identification of a variant of the α-GalA gene: c.611G > A (p.Trp204Ter). His father had a history of hypertrophic cardiomyopathy (HCM). Therefore, whole-exome sequencing of the pedigree was performed, and the results revealed an additional likely pathogenic MYH7 variant (c.1063G > A) (p.Ala355Thr). The final diagnoses included FD (classic), aHUS, and HCM (Fabry disease and MYH7 variants). Despite undergoing enzyme replacement therapy for FD, the patient’s renal function progressed to chronic kidney disease (CKD) stage 5, and there was no improvement in cardiac hypertrophy after 2 years. This case highlights the diagnostic challenges and complex management of patients with multiple rare disorders and a compounded genetic background.

## Introduction

Atypical hemolytic uremic syndrome (aHUS; OMIM:235400) is a thrombotic microangiopathy caused mainly by a dysregulated complement system. This disease is typically characterized by microangiopathic hemolytic anemia, thrombocytopenia, and end-organ damage, with the kidneys being the predominant organ affected. However, extrarenal manifestations such as stroke, seizures, and cardiovascular complications may also occur (1). Endothelial cell injury in aHUS is induced by pathological changes, such as dysregulation of the alternative complement pathway (complement-mediated HUS), mutations that cause loss of function of the lipid kinase diacyl glycerol kinase epsilon (DGKε), and recently identified TSEN2 mutations that disrupt tRNA biology (2). Complement-mediated HUS is caused by mutations in genes encoding complement factors, including complement factor H (CFH), factor I (CFI), factor B (CFB), C3, membrane cofactor protein (MCP), and thrombomodulin (THBD), or by autoantibodies against certain complement components (3). The prognosis for patients who present with aHUS is quite poor, with the first aHUS attack being associated with a mortality rate of approximately 25% resulting in end-stage renal disease requiring dialysis in approximately 50% of cases (4).

Fabry disease (FD, OMIM:301500) is an X-linked hereditary lysosomal storage disorder caused by mutation of the alpha-galactosidase A (*α*-GalA) gene, which leads to the misfolding and modification of the gene’s encoding product, *α*-GalA. Furthermore, a decrease in or absence of α-GalA activity can result in the progressive accumulation of globotriaosylceramide (Gb3) and the deacetylated derivative globotriaosylsphingosine (Lyso-Gb3) in tissues, which can lead to the involvement of multiple organs, including the heart, kidney, nerves, and skin (5). The exact incidence of FD is unknown, but it is estimated to be 1 in 100,000 individuals in the general population (6, 7). Hypertrophic cardiomyopathy (HCM; OMIM:192600) is the leading cause of sudden death in both adolescents and athletes. The prevalence of HCM is estimated to be at least 1/200 (8). HCM is primarily caused by mutations in genes that encode sarcomeric proteins, including MYH7, MYBPC3, TNNT2, TNNI3, MYL2, MYL3, TPM1, and ACTC1. In addition, some metabolic or systemic diseases, including amyloidosis, glycogen storage disease, Fabry disease, other lysosomal storage diseases, mitochondrial diseases, neuromuscular diseases, hemochromatosis, and dysplastic syndromes, may cause or be associated with left ventricular hypertrophy (8).

Here, we present an unusual case of atypical hemolytic uremic syndrome, Fabry disease, and hypertrophic cardiomyopathy, with a follow-up period of over 10 years.

## Clinical data

A 7-year-old boy with abdominal pain, vomiting, and edema was admitted to the hospital. Physical examination revealed hypertension (188/118 mmHg), edema in the abdominal wall, scrotum, and lower limbs, and hepatomegaly.

**Figure 1:**
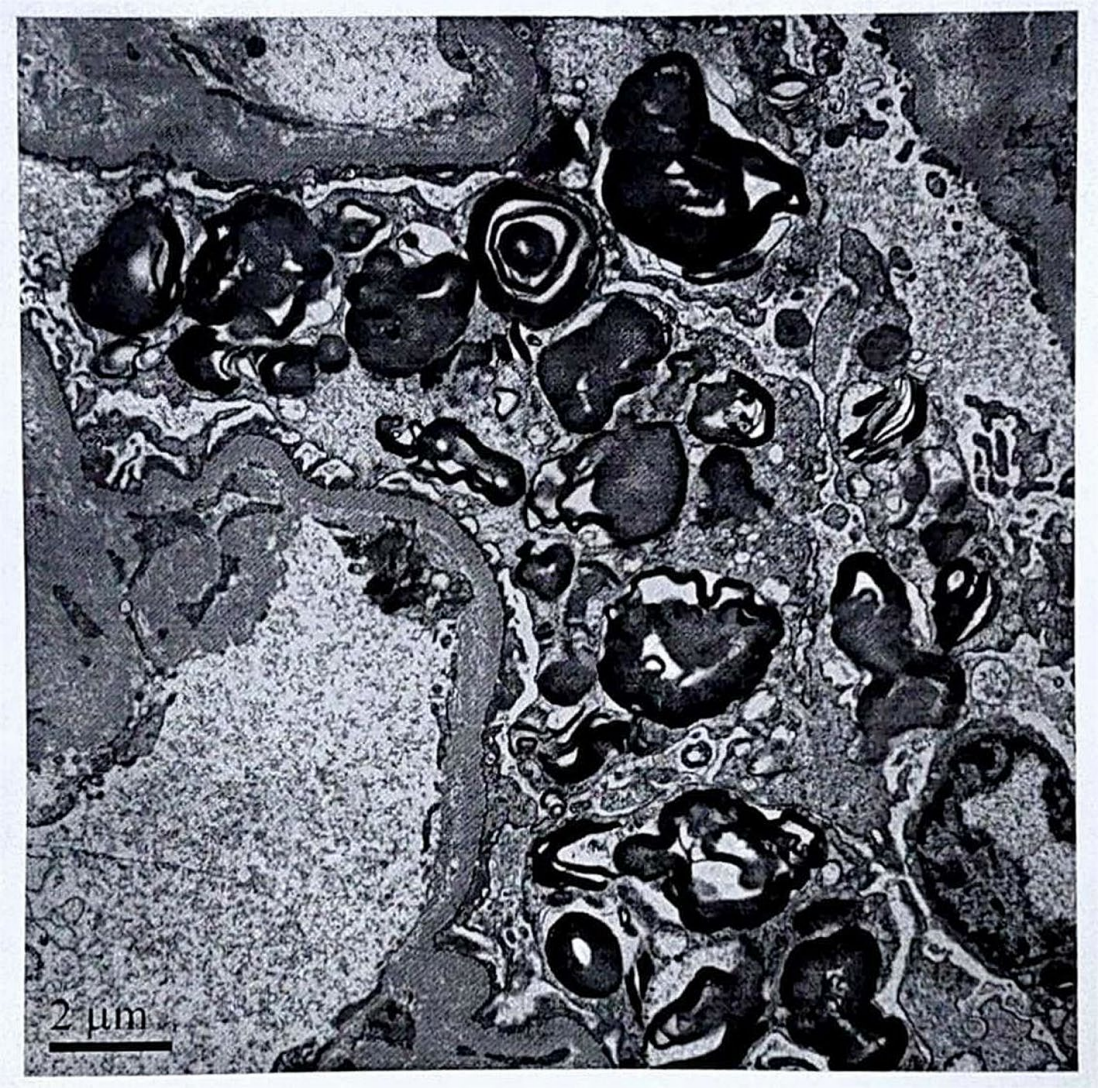
Electron microscopy revealed diffuse foot process fusion and myelin bodies in the cytoplasm of podocytes.

Laboratory investigations revealed microangiopathic hemolytic anemia (Hb 67 g/L; 3% schistocytes), thrombocytopenia (63 × 10^9^/L), acute kidney injury (serum creatinine 235.4 μmol/L), proteinuria (2.38 g/24 h), and low C3 levels (0.48 g/L). Coombs tests, antinuclear antibody (ANA) tests, vasculitis antibody tests, and infectious workups all yielded negative results. Bone marrow examination revealed hyperplastic anemia and thrombocytopenia. Electrocardiography revealed an incomplete right bundle branch block. Echocardiography revealed left atrial enlargement and slight thickening of all segments of the left ventricular myocardium. A renal biopsy showed slight proliferation in the mesangial region, collapse and ischemia with segmental necrosis, microaneurysmal dilation of the glomeruli, and “double-track” peripheral loops of some glomerular capillaries. The intima of the small arteries exhibited “onion skin” changes. Additionally, electron microscopy revealed diffuse foot process fusion and myelin bodies in the podocyte cytoplasm (Figure 1).

The boy was diagnosed with aHUS. At that time, eculizumab or ravulizumab was not yet available in mainland China. A methylprednisolone pulse (MPP) was administered, followed by cyclophosphamide and fresh frozen plasma infusion, along with antihypertensive and diuretic therapies (nifedipine, hydrochlorothiazide, furosemide, and fosinopril). The patient’s blood pressure gradually decreased to normal, the urine protein test results became negative, and the serum creatinine levels normalized. Steroids were discontinued after 1.5 years. However, 4 months later, the patient experienced proteinuria relapse with recurrent hypertension and was treated with prednisone, mycophenolate mofetil, and antihypertensive drugs. His urine protein levels ranged from negative to 1+.

At the age of 10, the patient experienced palpitations, shortness of breath, and acromioclavicular joint pain. Electrocardiogram (ECG) showed sinus arrhythmia, incomplete right bundle branch block, and T-wave changes. Cardiac ultrasound revealed left ventricular myocardial hypertrophy (LVH) (interventricular septal thickness 9.4 mm, left ventricular posterior wall thickness 9.7 mm, z score > 2.5, and left ventricular mass index 42.8 g/m^2.7^) and left ventricular outflow tract obstruction (LVOTO) with a gradient of 32 mmHg. Genetic testing was recommended, but the parents declined due to its high cost.

At the age of 14, he experienced finger numbness and toe pain accompanied by a fever. Treatments at local hospitals, including hydroxychloroquine, febuxostat, mecobalamin, valsartan, vitamin D3, folic acid tablets, and vitamin B6, were adopted but showed poor efficacy.

At the age of 17, the patient was hospitalized at another hospital because of aggravated toe pain. His serum creatinine concentration increased to 156 μmol/L, his estimated glomerular filtration rate (eGFR) decreased (38.4 mL/min·1.73 m^2^), and his *α*-GalA activity was low (< 1.00 μmol/L/h) (reference: 2.20–17.65 μmol/L/h). Genetic tests revealed an α-GalA c.611G > A (p.Trp204Ter) variant (pathogenic, FD, X-linked). The patient was subsequently transferred to our hospital. Physical examination revealed hypertension (153/91 mmHg) and diffuse small and large needle-tip rashes on the palms, fingertips, and scrotum. Further inquiry into his medical history revealed that the child had not experienced sweating since childhood, his maternal grandmother had a history of hand/foot pain, and his father had a history of HCM but tested negative for *α*-GalA mutation.

**Figure 2:**
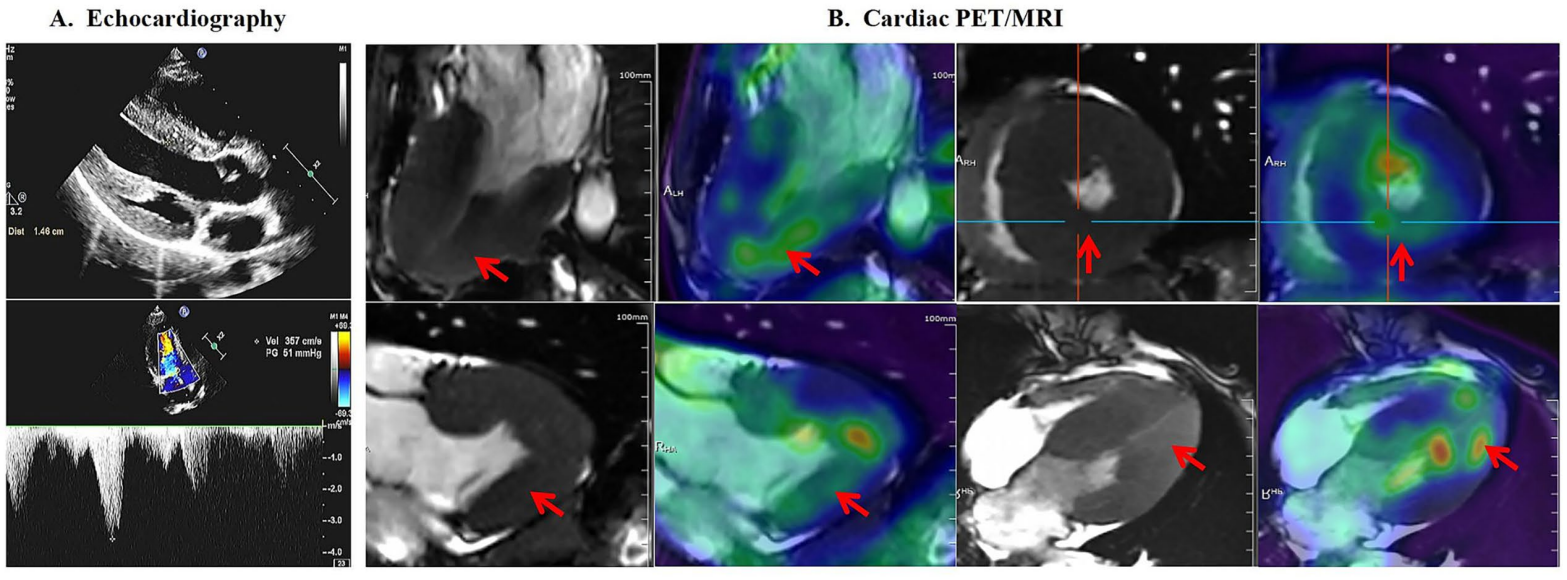
Echocardiography and cardiac positron emission tomography (PET)/magnetic resonance imaging (MRI) of the patient. **(A)** Echocardiography revealed left ventricular hypertrophy (interventricular septal thickness of 14.6 mm) and left ventricular outflow tract obstruction with a gradient of 51 mmHg after the squatting excitation test. **(B)** Cardiac PET/MRI revealed a diffusely thickened left ventricular wall and increased metabolism in the inferior and posterior walls (red arrows).

Further laboratory investigations revealed renal impairment (creatinine concentration of 138.30 μmol/L; eGFR of 43.3 mL/min/1.73 m^2^), proteinuria (1.01 g/24 h), left ear high-frequency hearing loss, and vortex corneal opacity. Repeated FD screening revealed a decreased *α*-GalA activity of 0.91 μmol/L/h and an increased Lyso-Gb3 concentration of 62.02 ng/mL (tandem mass spectrometry, reference: <1.11 ng/mL). Echocardiography revealed LVH (interventricular septal thickness 14.6 mm, left ventricular posterior wall thickness 10.8 mm, z score > 2.5, and left ventricular mass index 46.6 g/m^2.7^) and LVOTO with a gradient of 51 mmHg after the squatting excitation test. Cardiac positron emission tomography (PET)/magnetic resonance imaging (MRI) confirmed diffuse LV wall thickening with increased metabolic activity (Figure 2).

**Figure 3:**
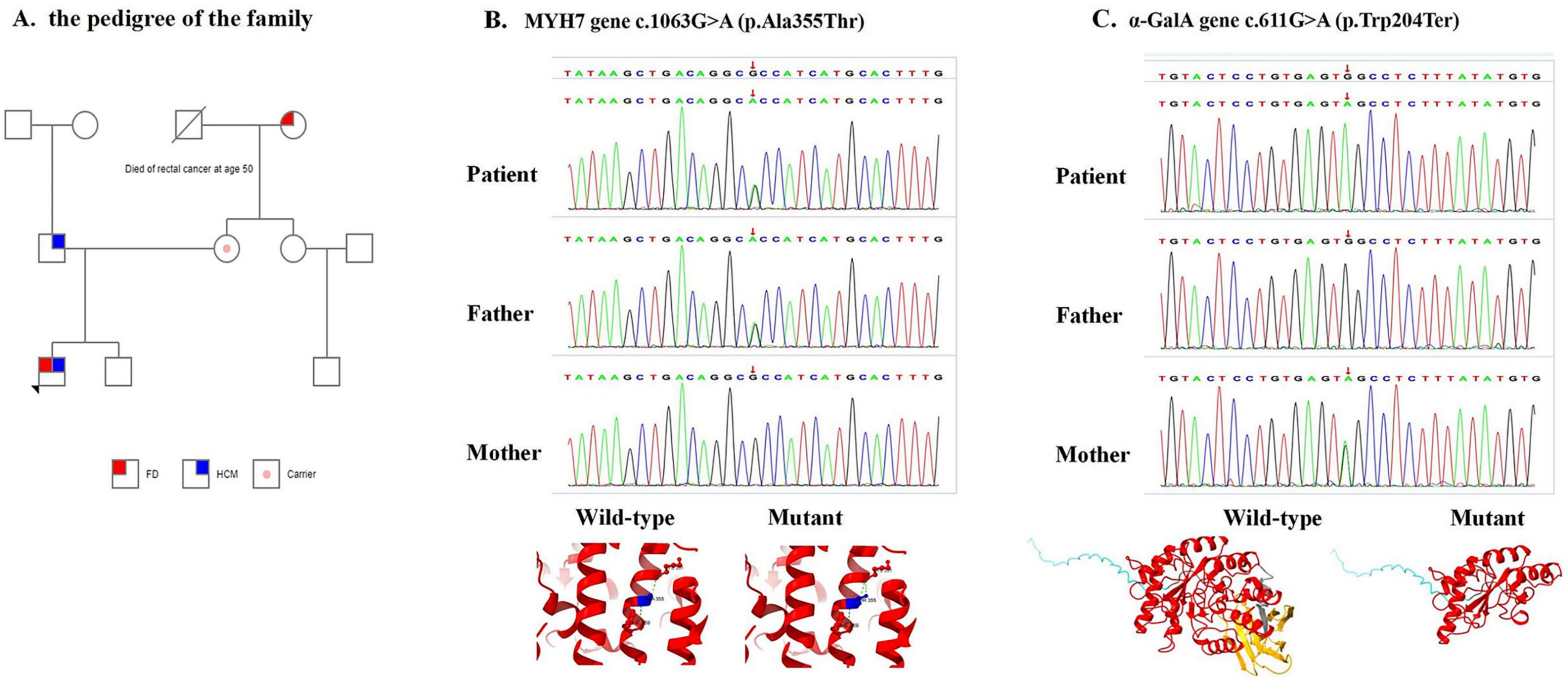
Pedigree and identification of the *MYH7* and *α*-GalA genes. **(A)** Pedigree analysis revealed that the father was affected by hypertrophic cardiomyopathy (HCM), the mother was a carrier of a gene variant associated with Fabry disease (FD), and the maternal grandmother was affected by FD. **(B)** Heterozygous mutation of the *MYH7* gene of the proband inherited from the father and predicted three-dimensional models of wild-type and mutant *MYH7* (p.Ala355Thr). **(C)** Heterozygous mutation of the α-GalA gene in the proband inherited from the mother and predicted three-dimensional models of wild-type and mutant α-GalA (p.Trp204Ter).

Given that his father had a history of hypertrophic cardiomyopathy but no *α*-GalA gene mutation, whole-exome sequencing was performed with the consent of the parents. Genetic testing revealed that the MYH7 gene c.1063G > A (p.Ala355Thr) variant (likely pathogenic, PS4 + PM1 + PM2 + PP2 + PP3, autosomal dominant) and the α-GalA gene c.611G > A variant (p.Trp204Ter) (pathogenic, PVS1 + PM1 + PM2 + PP3, X-linked) were inherited from the father and mother, respectively, according to the ACMG criteria (Figure 3). However, no pathogenic variants were detected in aHUS-related genes (CFH, CFI, CFB, C3, MCP, and THBD) or in the ADAMTS13 gene.

The final diagnosis for the patient was classic FD with aHUS (in remission) and HCM (associated with FD and the MYH7 variant). Enzyme replacement therapy (ERT) (agalsidase-*β* every 2 weeks) was initiated with valsartan and nifedipine for antihypertensive treatment and gabapentin for limb pain relief after a multidisciplinary consultation.

**Figure 4:**
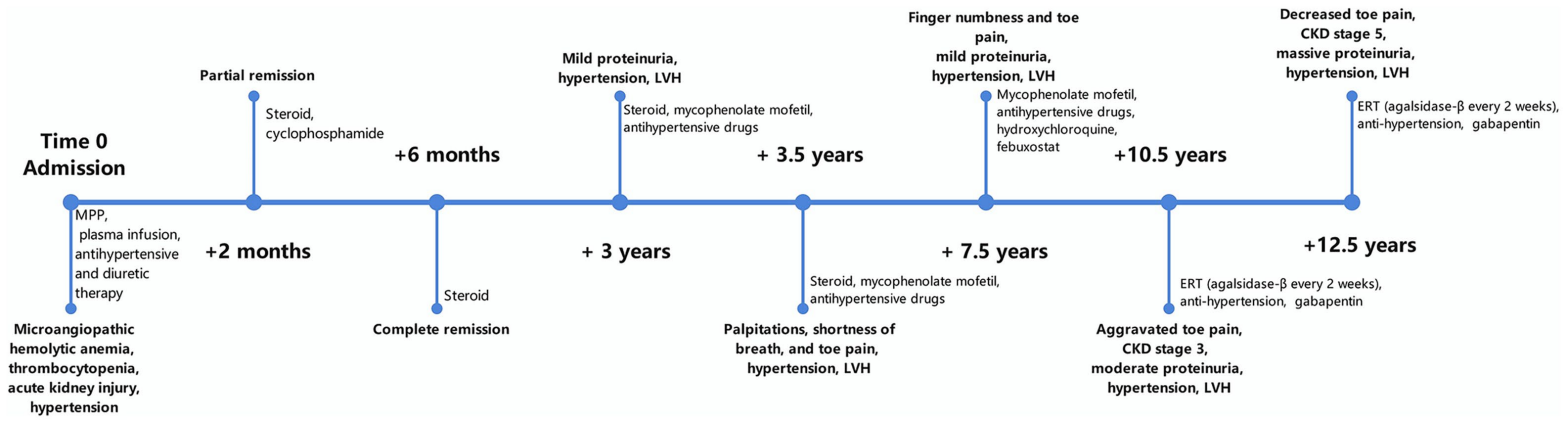
Timeline of the case. MPP, Methylprednisolone pulse; LVH, left ventricular myocardial hypertrophy; CKD, chronic kidney disease; ERT, enzyme replacement therapy.

After 2 years of ERT, the severity of his toe pain decreased significantly. His blood pressure ranged between 120 and 155/60–90 mmHg. However, his renal function progressively deteriorated to stage 5 chronic kidney disease (CKD) (serum creatinine concentration of 576 μmol/L and an eGFR of 10.4 mL/min/1.73 m^2^), and echocardiography revealed no significant alleviation of LVH (see Figure 4).

## Discussion

The patient was diagnosed with aHUS at the age of 7 based on microvascular hemolytic anemia, thrombocytopenia, acute kidney injury, and decreased blood complement C3 levels. He was treated with methylprednisolone pulse, cyclophosphamide, mycophenolate mofetil, and plasma infusion, and the symptoms were effectively controlled. At 12 years of age, the patient developed finger numbness and foot pain during a fever. At the age of 17, the patient presented with aggravated pain, renal impairment, and cardiac involvement. Screening for FD revealed decreased *α*-GalA activity and increased Lyso-Gb3 levels. Genetic testing revealed that the α-GalA c.611G > A (p.Trp204Ter) variant was inherited from the mother. Further examination revealed anhidrosis, cutaneous angioglioma, high-frequency hearing loss in the left ear, and corneal verticillata. Therefore, the patient was diagnosed with FD (classic). The patient’s maternal grandmother had a history of pain in both hands and feet, and his father had a history of HCM that manifested as chest pain. However, his mother was asymptomatic despite having the same pathogenic mutation in the *α*-GalA gene as the patient, possibly because of random inactivation of the X chromosome in women.

Myelin bodies were identified through electron microscopy of a renal biopsy when the patient was 7 years old. These myelin bodies are oval, parallel-layered lipid inclusions found in cellular lysosomes. These bodies can be found not only in FD patients, but also under other conditions, such as exposure to cationic amphiphilic drugs(hydroxychloroquine, chloroquine, amiodarone, and aminoglycosides), exposure to lithium, chromium and other heavy metals, and other diseases (light chain deposition disease, thin basement membrane nephropathy, coenzyme Q2 nephropathy) (9–11). The patient had neither a history of the aforementioned medications nor a history of heavy metal poisoning. However, the clinical manifestations and auxiliary examinations did not support these findings. The diagnosis of FD was not supported due to the lack of clinical symptoms of FD, the related family history, and the inability to detect *α*-GalA activity and Lyso-GL-3 at that time.

Both FD and aHUS are rare conditions, and cases of FD combined with HUS are very rare. Salvatore Coppola in Italy reported a case of a woman with aHUS who was treated with eculizumab. After maintaining hemodialysis, it was confirmed 15 years later that she was a carrier of the α-GalA c.352C > T (p. Arg118Cys) mutation by FD screening because of severe aortic and mitral valve insufficiency (12). In this case, the patient also developed aHUS, which was relieved after treatment with steroids and immunosuppressants. Ten years later, our patient was diagnosed with FD based on symptoms and FD screening. The pathogenesis of aHUS involves a deficiency in complement regulation, leading to excessive activation of the replacement pathway, capillary endothelial damage, and microthrombus formation in the microvascular lumen (4). FD is a type of small vessel disease, and GL3/Lyso-Gb3 deposition in endothelial cells can lead to vascular endothelial dysfunction. Chronic degradation of the endocalyx leads to endothelial dysfunction in FD, which can be partially reversed by FD-specific treatment (13). However, whether HUS is a rare manifestation of renal involvement in FD remains unclear and requires further investigation.

The etiologies of the kidney and heart involvement in this patient were complex. aHUS not only manifests as the classic triad of symptoms but can also lead to extrarenal manifestations such as heart failure. Some patients with aHUS experience progressive hypertension, proteinuria, and progressive elevation in creatinine levels as recurrent symptoms after remission. FD patients may exhibit renal manifestations such as proteinuria and decreased glomerular filtration rate (GFR), as well as cardiac manifestations such as left ventricular hypertrophy and heart failure (14). Left ventricular hypertrophy in FD is mostly centripetal, whereas in a few cases, it is asymmetrical and eccentric (15). In this case, repeated echocardiography revealed that the degree of myocardial hypertrophy gradually increased, which seemed to be explained by the diagnosis of FD. However, the patient’s father had a history of hypertrophic cardiomyopathy and did not carry an *α*-GalA gene mutation. Renal insufficiency is a common complication in patients with HCM, particularly in women, older individuals, and those with abnormal left ventricular systolic function. Therefore, genetic testing was critical for the diagnosis of this patient.

In this case, whole-exome sequencing revealed the variants of MYH7 c.1063G > A (p.Ala355Thr) and α-GalA c.611G > A (p.Trp204Ter,226). No mutations in aHUS-related genes or in the ADAMTS13 gene were detected. Previous reports have revealed that familial HCM could be caused by MYH7 c.1063G > A (p.Ala355Thr), and carriers may present with complete left bundle branch block, left ventricular hypertrophy, atrial fibrillation, and asymmetric obstructive hypertrophic cardiomyopathy (16). Patients with FD carrying the *α*-GalA c.611G > A (p.Trp204Ter,226) variant may also present with left ventricular myocardial hypertrophy (17, 18). Therefore, cardiomyopathy in our patient may have resulted from the coinfluence of the MYH7 c.1063G > A (p.Ala355Thr) and α-GalA c.611G > A (p.Trp204Ter,226) variants.

The final diagnoses of the child were aHUS, FD (classic), and HCM (FD associated with the MYH7 variant). The severity of aHUS in this patient decreased after the treatment with steroids, immunosuppressants, and plasma therapy, and no related genetic variants were found. Therefore, subsequent eculizumab treatment was not administered. ERT was initiated after joint consultation and evaluation by experts in pediatric and adult nephrology, pediatric and adult neurology, cardiology, cardiac ultrasonography, otolaryngology, and genetics. Although ERT and chaperone therapy are effective in FD patients, other treatments such as ACE inhibitors, angiotensin receptor blockers, and SGLT2 inhibitors can also provide nephroprotective effects when renal damage is established (19). Valsartan was administered to this patient to control hypertension and alleviate proteinuria.

However, after 2 years of ERT treatment, the patient’s renal function progressively deteriorated, reaching stage 5 CKD, with no alleviation of left ventricular hypertrophy. The poor renal and cardiac response to ERT may be attributed to the following factors: (1) advanced, irreversible organ damage at ERT initiation; (2) compounding pathogenic effects of dual (MYH7 and *α*-GalA) genetic mutations on cardiomyopathy, potentially limiting ERT efficacy on cardiac structure; and (3) potential interactions between the vascular endothelial injury pathways of aHUS and FD, complicating the disease course.

## Patient perspective

From the patient’s perspective, the journey through diagnosis and treatment was fraught with uncertainty and challenges. The initial relief experienced after aHUS treatment with steroids, immunosuppressants, and plasma therapy was tempered by the subsequent discovery of FD and HCM. The decision to initiate ERT was a beacon of hope; however, the lack of significant improvement in renal function and the alleviation of left ventricular hypertrophy over 2 years of treatment cast doubt on its long-term efficacy. The patient and their family had to navigate the emotional and physical toll of frequent hospital visits, medical tests, and the side effects of various medications. Progression to CKD stage 5 was a significant setback, altering the patient’s quality of life and future prospects. Persistent LVH despite ERT raised additional concerns regarding the potential for heart failure and other cardiovascular complications.

Throughout this ordeal, the patient and their family sought support from medical professionals, patient advocacy groups, and fellow patients. They learned to cope with uncertainty by focusing on symptom management and striving for the best possible quality of life. The importance of genetic counseling and testing has become evident, providing a clearer understanding of the underlying causes of the patient’s conditions and guiding future treatment decisions.

## Conclusion

This case highlights the challenges of diagnosing and managing patients with multiple rare genetic disorders, particularly those with overlapping clinical features. It also emphasizes the importance of a comprehensive diagnostic workup, including genetic testing, in patients with atypical presentations or a family history suggestive of inherited diseases. It underscores the challenges in managing patients with concurrent rare diseases, where treatment for one condition may not address the issues caused by another independent genetic etiology.

### Limitations

Genetic testing related to aHUS was not performed during the initial presentation due to limited resources. The follow-up period after ERT initiation (2 years) was relatively short for assessing long-term outcomes in patients with FD. Functional studies to characterize the interactions between the identified MYH7 and *α*-GalA variants were not conducted.

## Data Availability

The original contributions presented in the study are included in the article/Supplementary material, further inquiries can be directed to the corresponding author.
