# Supplementary material for: An unusual trilogy: a case of comorbid aHUS, Fabry disease, and hypertrophic cardiomyopathy
Source: Front Med (Lausanne). 2026 Jan 30;13:1700541. doi: 10.3389/fmed.2026.1700541 (PMC12900690; doi:10.3389/fmed.2026.1700541)
Supplement: Supplementary file 1 [file Table_1.DOCX]

**Table 1 Patient biochemical and clinical data at baseline and follow-up**

|  | **Time 0** | **+2 months** | **+6 months** | **+3 Years** | **+3.5 Years** | **+7.5 Years** | **+10.5 Years** | **+12.5 Years** |
| --- | --- | --- | --- | --- | --- | --- | --- | --- |
| Blood WBC(x10^9/L) | 6.15 | 11.26 | 7.1 | 8.74 | 4.27 | 4.74 | 6.68 | 5.45 |
| Hemoglobin(g/L) | 67 | 110 | 126 | 164 | 124 | 166 | 130 | 103 |
| Blood platelet(x10^9/L) | 63 | 387 | 226 | 232 | 137 | 276 | 194 | 210 |
| Urinary protein | 2+ | +- | - | +- | - | -~2+ | 2+ | 2+ |
| Urinary RBC(/HP) | 61 | 5.3 | 2.4 | 1.3 | 3.2 | 4.1 | 2 | 1.3 |
| 24-h urinary protein content(g) | 2.38 | ND | ND | 0.27 | 0.01 | 0.25~0.61 | 1.01 | 5.5 |
| Blood urea nitrogen(mmol/L) | 40.4 | 13 | 9 | 8.3 | 6.5 | 7.9 | 14 | 36.3 |
| Blood creatinine(µmmol/L) | 235.4 | 107 | 75.6 | 64 | 71 | 95 | 138.3 | 576 |
| eGFR (ml/min/1.73 m²) | 18 | 40 | 62 | 75 | 69 | 62 | 43.3 | 10.4 |
| LVMI (g/m^2.7^) | 42.1 | ND | ND | 36.6 | 42.8 | ND | 46.6 | 48.2 |

eGFR: estimated glomerular filtration rate, LVMI: left ventricular mass index, ND: no data.
